# Utilization of a mobile phone application to increase access to sexual and reproductive health information, goods, and services among university students in Uganda

**DOI:** 10.1186/s12978-020-01037-z

**Published:** 2021-05-17

**Authors:** Robinah Nalwanga, Elly Nuwamanya, Afra Nuwasiima, Janet U. Babigumira, Francis T. Asiimwe, Joseph B. Babigumira

**Affiliations:** 1 GHE Consulting, P.O Box 27011, Kampala, Uganda; 2 Department of Community Medicine and Public Health, Sahlgrenska Academy, University of Gothenburg, P. O Box 414, 40530 Gothenburg, Sweden; 3 Saw Swee Hock School of Public Health, National University of Singapore , Block MD1, 12 Science Drive 2, Tahir Foundation Building, #09-03M, Singapore, 117549 Singapore

**Keywords:** Sexual and reproductive health, Students, Utilization, Services, Kyambogo University

## Abstract

**Background:**

Innovations to increase access to sexual and reproductive health (SRH) information, goods, and services are needed, particularly in low-income settings. This study assessed the utilization of a mobile phone application (MPA) to increase access to SRH information, goods, and services among university students in Uganda.

**Methods:**

We conducted a cross-sectional analysis of data from: (1) an endline survey performed as a consequence of a randomized controlled trial (RCT) of the effectiveness of the MPA, and (2) data from use of the MPA for accessing information, goods, and services over the 6-month time period of the RCT, obtained from in-MPA data collection service providers. We performed descriptive analysis of participant characteristics and their association with the utilization of the MPA using logistic regression; analyses of MPA use for accessing different types of information, goods, and services by gender; and analyses of functionality attributes of the MPA and related services.

**Results:**

In the study population of young (median 22 years) predominantly female (61%) students, the utilization of the MPA by those who downloaded it was high (81% overall, 82% female, and 82% male). The most popular information portal was the frequently asked questions (71% utilization); the most popular goods were condoms for males (77% utilization) and sanitary pads for females (94% utilization); and the most popular service was HIV testing and counseling (60% utilization). The MPA demonstrated predominantly positive (responsiveness, non-distracting in-app advertisements, and ease of use) attributes.

**Conclusion:**

A mobile phone app to increase access to SRH information, goods, and services among university students in Uganda demonstrated high utilization. The results of this study support ongoing and future technical improvement efforts and research on effectiveness, economic efficiency, and scalability, along the continuum of activities to scale this intervention in order to improve SRH in low-income settings.

**Trial Registration:**

MUREC1/7 No. 07/05-18. Registered; June 29, 2018.

## Background

Uganda has an age distribution typical of low-income, high fertility, and low life-expectancy nations: approximately 78% of its population is below 30 years of age [1]. In low-income settings, young adults, particularly girls, experience biological, psychological, and social changes that may predispose them to risky sexual behaviors such as early sex experimentation, unprotected sex, sex for money, sex under the influence of alcohol or drugs, and multiple sexual partners [1, 2]. Additionally, young adult women usually lack awareness of these risks, face social stigma, are often unemployed, with limited access to sexual and reproductive health (SRH) information, goods, and services [2, 3]. This puts them at further risk of SRH health problems such as early and unplanned pregnancies, unsafe abortions, and sexually transmitted infections (STIs), including HIV/AIDS.

Given such a large proportion of young adults relative to other adults in the Ugandan population, youth-friendly services, including SRH services, should be a public policy priority. SRH services ensure that individuals have access to accurate SRH information and high-quality, accessible, and affordable SRH goods and services. In addition to socio-economic benefits, the universal provision of SRH services has direct health outcomes benefits. For instance, universal access to modern contraceptives has the potential to prevent up to 30% of the maternal and 10% of the child deaths, reduce STIs, and improve the quality of life among adolescent girls [4].

Although there have been improvements, knowledge, awareness, and uptake of SRH information, goods, and services in low-income countries is low in general, and more so for young adults [5, 6]. Barriers to increased access include lack of confidentiality and privacy, cultural and societal discrimination, poverty, and long distances between homes and health facilities [3, 7–9]. Consequently, there is a need for novel SRH interventions to increase the access that is acceptable to young adults and that fit their lifestyles.

Because of the increase in cellular network reach and reduced costs of mobile phones, as high as 90% of individuals in high-, middle-, and low-income countries now have mobile handsets [10]. In Uganda, despite relatively high costs of voice and data services, nearly 71% of Ugandans own mobile phones, and the coverage of internet-enabled handsets is increasing [11]. Therefore, digital health interventions (DHIs), including mobile health (mHealth), offer a potential avenue to increase access in this population. The use of mHealth to increase awareness and access to SRH goods and services is increasingly popular around the world [12–14] and leverages the key youth-friendly attributes of confidentiality and privacy [15], as well as duo entertainment and information value. And increasingly, mobile phone applications (MPAs) are being used to back up media campaigns for short-term behavioral change, although sustainability has not been achieved [14, 16].

MHealth for increased access to SRH services has shown the potential for acceptability and feasibility in the context of contraception [17], medical abortion [18], and maternal health during pregnancy [19, 20]. A study in Bangladesh in which the median age was 29 years of age found high acceptability but low utilization of mHealth services due, in part, to a perception that they would prevent in-person consultations for SRH [15]. Given their ability to extend services beyond voice and text messaging, DHIs that are internet-based, such as MPAs, have the potential to further increase access to SRH services. Although some studies have shown moderate to high acceptability of MPAs and MPA-driven SRH interventions [21–23], we found none that were conducted in low-income settings. Additionally, on reviewing the literature, we found multiple studies on the utilization of MPAs for access to contraception information [24, 25], but none that were goods and services focused.

In this study, we assessed the utilization of a MPA to increase access to SRH information, goods, and services among university students in Uganda. The study was a part of a broader MPA development and impact evaluation project conducted over an 18-month period [26].

## Methods

### Study setting

The study was conducted at Kyambogo University (KYU) in Kampala, Uganda. KYU, the second-largest university in the country, is situated in an urban area and has a student population of over 51,000 students [26]. KYU students have a near-universal mobile phone penetration and at least 50% smartphone penetration.

### Intervention description

The study was one of five studies performed as part of an extensive project to develop, pilot, and evaluate a MPA to increase uptake of SRH information, goods, and services by university students in Uganda. The project was conceived by GHE Consulting, who also coordinated study activities and led the impact evaluation. The MPA was developed by the tech firm Gershom Technologies and pilot-tested by a consortium including GHE Consulting (https://www.gheuganda.com/); three health facilities operating near KYU—Kyambogo Medical Centre (public health facility), Alpha Medical Centre, and Nim Pharmacy (private health facility and pharmacy, respectively); SafeBoda (https://safeboda.com/ug/), a transportation company; Beyonic (https://beyonic.com/), a payments company; and the Reproductive Health Division of Uganda’s Ministry of Health.

A detailed description of the development of the MPA will be published elsewhere, and a protocol for the impact evaluation has been published [26]. Briefly, the MPA was developed in the Android operating system and archived on the Google Play store. The app was designed to link the different goods and services providers: health facilities which provided SRH goods and services; Beyonic, which managed payments and co-payments; SafeBoda which provided transportation of ordered goods and app users to receive services; and GHE Consulting for coordination, management, including the rolling out of discounts, which ranged from 50 to 100% (leveraged on the normal market prices), and oversight.

The app included the following features: (1) sign-up and sign-in; (2) SRH product delivery platform for making orders for goods (sanitary pads, condoms, contraceptives (pills and injections), pregnancy tests, and pain killers), (3) Online booking and appointments module for SRH services (HIV testing and counseling, STI diagnosis and treatment, family planning counseling, and general SRH consultation); (4) an SRH information module (menstrual period tracker, frequently-asked questions (FAQs), SRH tips, and a live chat); (5) a payments module to enable provider payments by GHE Consulting, co-pays by clients, and payments for transportation; (6) a delivery module to enable clients to track shipments, set up pickups for in-facility visits, and set up pick up points for products; and (7) a security module for authentication and password protection. Embedded in the app was an advertising interface; the app was designed to test potential sustainability at scale through in-app advertising. While the study provided a major subsidy for goods and services, there was a modest co-pay that was paid by app users through a link between mobile money and the Beyonic payment system. The co-pay was designed to test the utility of client payments in potential future iterations of the app after the pilot period.

### Study design

The study was a one-year pilot project with two complementary sub-studies; (1) a randomized control trial (RCT) to evaluate the effectiveness of the MPA to increasing access to SRH information, goods, and services among KYU students [27], (2) a cross-sectional study to assess the acceptability and usability of MPA. The acceptability study was part of the baseline survey used during data collection. The inclusion criteria for the extensive study (RCT) were KYU students 18 to 30 years of age, self-reported sexual activity in the last six months, more than 12 months to graduation, consent, and access to an internet-enabled Android smartphone [27]. The utilization study used data collected from MPA users over the 6-month period of the RCT from in-app data collection and data provided by the healthcare providers. All participants who downloaded the MPA were considered in the utilization analysis.

### Recruitment and procedure

Participants were randomly recruited from the university halls and private affiliated hostels at KYU. A list of university halls and affiliated hostels and their estimated population was prepared with the help of student leaders at the university, and permission to access the halls and hostels was sought from the respective wardens. Trained research assistants moved to the selected rooms and recruited the study participants. Recruited participants were first screened for eligibility before enrollment in the study. A standard questionnaire was administered to consenting participants at enrollment. Enrolled participants were then randomized to two arms of the study, participants that were supposed to receive the app and control participants. We recorded the telephone number of the consenting participant, link this to a unique study ID, and passed this on to the app developer. The app developer used a computer-generated random sequence of numbers matched to telephone numbers and study ID to allocate participants to the trial. The app was made available for download from the developer’s repository, and it was password protected. The research team at GHE was blinded to the allocation sequence. Participants randomized to receive the app received text messages with the app link and guidelines on how to install the app on their phones. These further received more guidelines on how the app was used and its benefits. Enrolled study participants were followed up at one and six months to determine the effectiveness of the SRH app in increasing SRH awareness and uptake*.* At the close of the study (month six), we conducted a user satisfaction survey among participants in the intervention arm of the study.

### Sampling and sample size

We created a sampling frame of rooms in all halls of residence and affiliated hostels at KYU with the help of student leaders and hall wardens. We randomly selected a pre-calculated sample of 1,086 rooms and selected one room occupant per room (using a random draw to choose respondents in rooms with multiple occupants) and assigned half to the intervention group and half to the control group. The utilization study was conducted on the randomized participants to the intervention group and thereby received a recommendation to download the MPA and an access code to operationalize it.

### Measurement of study outcomes

We measured utilization as the number and proportion of eligible participants that used the MPA to access SRH goods and services in a six-month follow-up period. Individuals that did not utilize the MPA were probed for reasons, and categories of reasons for non-utilization were created for the analysis. We also collected data on types of information, goods, and services used and the functionality of the MPA and related services. We also asked users to provide information on their perceptions of the potential benefits of the MPA and areas of potential improvement of the MPA experience.

### Data collection

We used data from multiple sources to assess the utilization of the MPA. The end-line survey administered as part of the RCT included questions on demographic characteristics and willingness to download the MPA, including the reasons for refusal. We obtained utilization data by using the administrator module of the MPA to download data and validating these data at the three health facilities where goods and services were provided. We recruited and trained research assistants on the rationale and methods of the study and on the responsible and ethical conduct of research. We also provided training on Android-phone open data kit (ODK), which was used to collect the data.

### Data analysis

We performed the analysis in Stata 13 (Stata Corporation, College Station, TX). We performed descriptive analyses of participant characteristics using means (continuous variables) and proportions (categorical variables) and bivariate analyses using chi-square tests to assess the characteristics of participants by utilization. We then assessed the association between different variables and the two outcome variables (utilization) using univariate and multivariate logistic regression. We reported both unadjusted and adjusted odds ratios with p-values and 95% confidence intervals (CIs). We considered all results significant at the 5% level.

### Ethical considerations

We obtained ethical clearance from the Mbarara University of Science and Technology ethics review committee and the Uganda National Council of Science and Technology. We also received regulatory clearance from the Ministry of Health and KYU. We administered and received informed consent from all participants.

## Results

### Recruitment and randomization

**Fig. 1:**
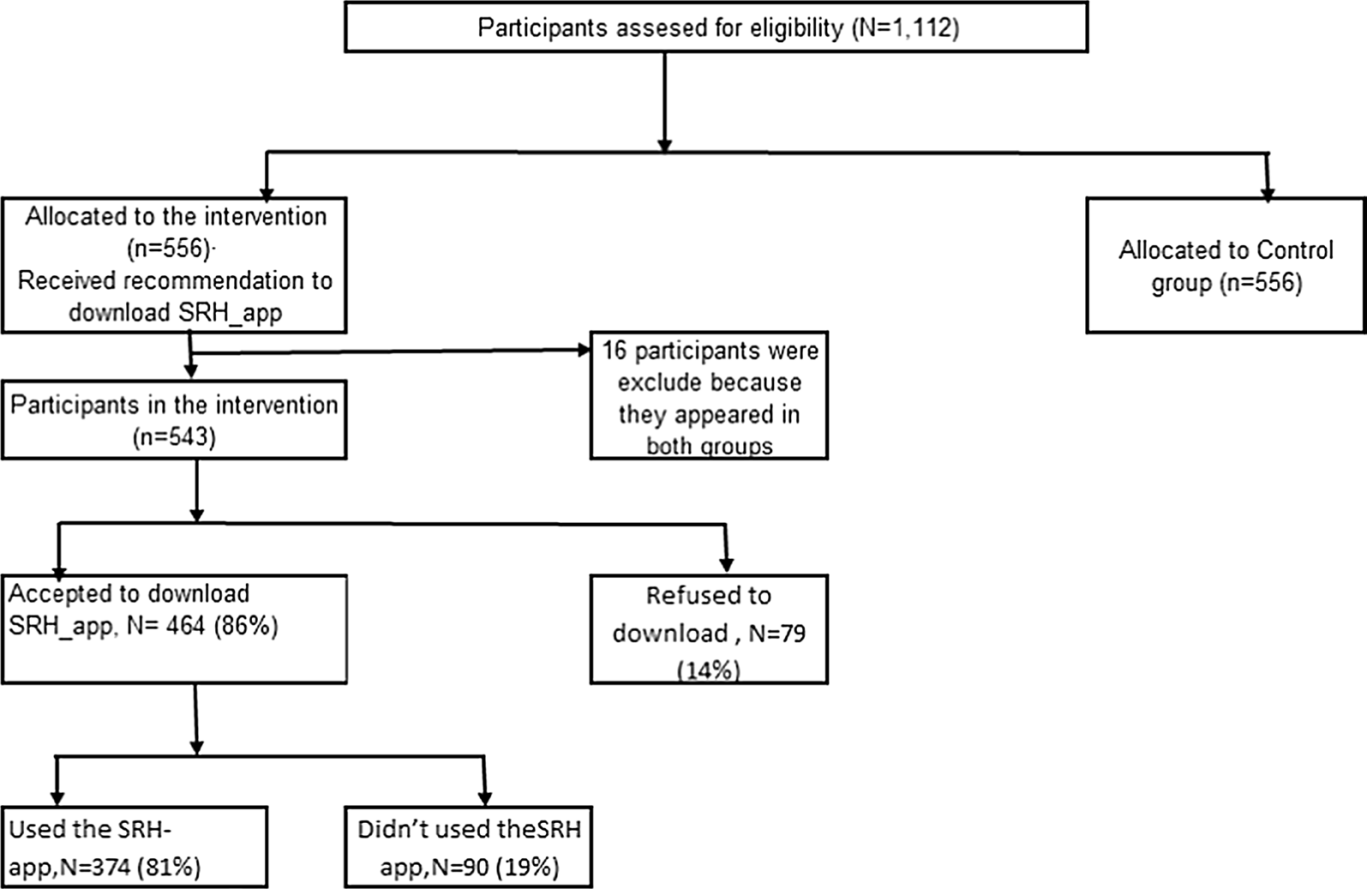
Consort diagram

Figure 1 shows that a total of 1112 participants were enrolled in the study. Study participants were further randomized into two groups, i.e., the intervention and control group. During the analysis, we discovered that 16 participants were allocated to both groups (they used different phone numbers), and we exclude them from the analysis. This resulted in 543 participants in the intervention group and 556 participants in the control group.

### Participant characteristics

The median age of students was 22 years, and most students were female (61%), Christian (90%), had urban (42%) or peri-urban (33%) hometowns, were in their second year of study (38%), were jobless (86%), and were tuition sponsored by parents or guardians (72%). Table 1 shows the demographic characteristics of the participants.

**Table 1:** Baseline characteristics by participation

Characteristics	All, n (%)	Downloaded, n (%)	Refused, n (%)	P-value
Age (median (IQR), [range])	22 (21, 23) [18,30])	22 (21, 23) [18,30])	22 (21, 23) [18,30])	
Gender				0.389
Male	210 (38.7)	176 (37.9)	34 (43.0)	
Female	333 (61.3)	288 (62.1)	45 (56.9)	
Residence				0.888
Hall on campus	135 (24.9)	116 (25.0)	19 (24.1)	
Off-campus hostel	294 (54.1)	248 (53.5)	46 (58.2)	
Rental home	101 (18.6)	88 (18.9)	13 (16.5)	
Own home	02 (0.37)	02 (0.4)	00 (0.0)	
Parent's home	11 (2.03)	10 (2.2)	01 (1.3)	
Home town				0.684
Urban	229 (42.2)	196 (42.5)	32 (40.5)	
Peri-urban	177 (32.6)	148 (31.9)	29 (36.7)	
Rural	137 (25.2)	119 (25.7)	18 (22.8)	
Marital status				0.928
Dating	298 (54.9)	255 (54.9)	43 (54.4)	
Single	225 (41.4)	191 (41.2)	34 (43.0)	
Cohabiting	14 (2.6)	13 (2.8)	01 (1.3)	
Married	04 (0.7)	03 (0.7)	01 (1.3)	
Divorced	01 (0.2)	01 (0.2)	0 (0.0)	
Widow/widowed	01 (0.2)	01 (0.2)	0 (0.0)	
Year of study				0.144
Year 1	170 (31.3)	140 (30.2)	30 (37.9)	
Year 2	205 (37.8)	173 (37.3)	32 (40.5)	
Year 3	142 (26.2)	130 (28.0)	12 (15.2)	
Year 4	24 (4.4)	19 (4.1)	05 (6.3)	
Year 5	02 (0.37)	02 (0.4)	0 (0.0)	
Employment				0.512
None	464 (85.5)	399 (85.9)	65 (82.3)	
Paid employee	46 (8.47)	36 (7.8)	10 (12.7)	
Volunteer	01 (0.18)	01 (0.2)	0 (0.00)	
Self-employed	32 (5.9)	28 (6.0)	04 (5.7)	
Tuition source				0.78
Parent/guardian	391 (72.0)	322 (71.5)	59 (74.7)	
Self	20 (3.7)	18 (3.9)	02 (2.5)	
Government	104 (19.2)	88 (18.9)	16 (20.3)	
Scholarship	27 (4.9)	25 (5.4)	02 (2.5)	
Others	01 (0.2)	01 (0.2)	0 (0.0)	
Religion				0.631
Christian	487 (89.7)	418 (90.1)	69 (82.3)	
Muslim	43 (7.9)	36 (7.8)	07 (8.9)	
Others	13 (2.4)	10 (2.2)	03 (3.8)	

### Utilization

Of 543 students who received a recommendation to download the MPA, 464 (86%) accepted and downloaded the app. Of the 210 males, 176 (84%) accepted, and of 333 females, 288 (86%) accepted to download the SRH-MPA.

Of the 464 students that downloaded the MPA, 374 (81%) used it to access SRH information, goods, and services over six months. The proportion of MPA users was similar by gender: 235 of 288 (82%) of females and 139 of 176 (82%) of males used the MPA to access SRH goods and services over 6 months. There were no significant differences in MPA utilization by other participant characteristics. Results further indicated that out of 464 students who downloaded the app, 90 students did not use the app. Some participants cited more than one reason for not using the MPA, and the distribution of their reasons was as follows: slow internet speed and denied access to the MPA—55 students (34%); poor app functioning—33 students (20%); difficulty in app installation—24 students (15%); lost phone—13 students (4%); and other reasons—8 students (2%). Another 30 students (8%) did not cite a reason and simply did not use the MPA.

The study participants proportionately used the MPA more than once to access SRH information, goods, and services. The distribution of frequency of MPA use was as follows: daily (6%), at least weekly (30%), at least monthly (40%), and at least once in 6 months (23%). Table 2 is an inventory of the use of SRH information, goods, and services use by app users by gender. The most information portal was the FAQs: 71% overall and equal by gender. The most popular good was condoms for males (77%) and sanitary pads for females (66%). The most popular service was HIV testing: 60% overall, 55% for males and 63% for females.

**Table 2:** Types of SRH information, goods, and services accessed by the study participants

	Male (n (%))	Female (n (%))	All (n (%))
Information			
Period tracker	0 (0.00)	113 (48.9)	113 (31.6)
FAQs	97 (71.3)	163 (70.6)	260 (70.8)
SRH tips	78 (57.6)	127 (54.9)	205 (55.9)
Live chat	11 (8.1)	28 (12.2)	39 (10.7)
Goods			
Sanitary pads	23 (16.6)	222 (94.5)	245 (65.5)
Condoms	104 (76.5)	11 (4.8)	115 (31.3)
Contraceptives^a^	13 (9.6)	33 (14.3)	46 (12.5)
Pregnancy tests	05 (3.7)	29 (12.6)	34 (9.3)
Pain killers	0 (0.0)	01 (1.5)	1 (0.5)
Services			
HIV testing and counseling	76 (54.7)	147 (62.6)	223 (59.6)
STI testing and treatment	44 (31.7)	169 (71.9)	213 (56.9)
Family planning counseling	0.0 (0.0)	13 (5.5)	13 (4.8)
SRH consultation	39 (28.1)	53 (22.6)	92 (24.6)

*FAQs* frequently-asked questions, *SRH* sexual and reproductive health, *HIV* human immunodeficiency virus, *STI* sexually-transmitted infections

^a^Included pills, emergency contraception, injectable contraceptives, and intrauterine devices

Table 3 is a summary of the data on the functionality attributes of the MPA by gender. More than half (55%) of students reported responsiveness by providers to orders for goods and services. Less than half (45%) of the students reported prompt delivery of goods or pick up for services. A majority (81%) of students reported receiving a significant discount on goods and services. Similarly, a majority (65%) of students reported ease of payment of copays for goods and services. Most students (75%) reported that in-app adverts were not distracting. A majority of students (77%) found the MPA very easy or easy to use, and 86% of students found the in-app instructions very easy or easy to follow. There were no statistically significant differences in functionality attributes except in perception of a substantial discount for goods, services, and transportation in which a significantly larger proportion of women (95%) than men (83%) perceived a substantial discount on SRH goods and services.

**Table 3:** Functionality attributes of the mobile phone application by gender

Attribute	Male, N (%)	Female, N (%)	All, N (%)	P-value
Clinics’ responsiveness				
Responsive	66 (47.5)	140 (59.6)	206 (55.1)	0.114
Somewhat responsive	31 (22.3)	36 (15.3)	67 (18.4)	
Neutral	11 (7.9)	21 (8.9)	32 (8.6)	
Somewhat unresponsive	09 (6.5)	15 (6.4)	24 (6.2)	
Unresponsive	22 (15.8)	23 (9.8)	45 (12.0)	
Delivery speed				
Fast	57 (41.0)	111 (47.2)	168 (44.9)	0.37
Neutral	53 (38.1)	70 (29.8)	114 (31.9)	
A little late	16 (11.5)	26 (11.1)	42 (11.2)	
Very late	13 (9.4)	28 (11.9)	41 (11.0)	
Substantial discount				
Discounted good or service	99 (71.2)	204 (86.8)	303 (81.0)	0.001*
Discounted transportation	16 (11.5)	14 (6.0)	30 (8.1)	
No discount	24 (17.3)	17 (7.2)	41 (11.0)	
Ease of payment				
Easy	79 (56.8)	163 (69.4)	242 (64.7)	0.079
Neutral	38 (27.3)	42 (17.9)	80 (21.4)	
Somewhat difficult	12 (8.6)	19 (8.1)	31 (8.3)	
Very difficult	5 (7.2)	11 (4.7)	21 (5.6)	
Experience of in-app ads				
Very distracting	02 (1.4)	04 (1.7)	06 (1.6)	0.225
Somewhat distracting	05 (3.6)	07 (3.0)	12 (3.2)	
Neutral	08 (5.8)	10 (4.3)	18 (4.8)	
Minor distraction	28 (20.1)	26 (11.9)	56 (15.0)	
Not distracting at all	96 (69.1)	186 (79.2)	282 (75.4)	
Ease of use of app				
Very easy	68 (48.9)	120 (51.1)	188 (50.3)	0.915
Easy	36 (25.9)	63 (26.8)	99 (26.5)	
Neutral	13 (9.4)	18 (7.7)	31 (8.3)	
Difficult	22 (15.8)	34 (14.5)	56 (15.0)	
Ease of in-app instructions				0.503
Very easy	62 (43.2)	106 (45.1)	166 (44.4)	
Easy	64 (45.3)	91 (38.7)	154 (41.2)	
Neutral	05 (3.6)	13 (5.5)	18 (4.8)	
Difficult	11 (7.9)	25 (10.6)	36 (9.6)	

*Denotes significance at the 5% level

The following was the distribution of the dominant themes as provided in comments on potential benefits of the MPA and areas of potential improvement of the MPA experience among 80 students that provided information: 24 students (30%) thought that the MPA warranted widespread dissemination to students; 18 students (23%) thought the MPA would be beneficial to students; 14 students (18%) thought that improvements to the MPA’s functionality were needed; 13 students (16%) thought that the improvements were needed on health provider responsiveness; 9 students (11%) thought that MPA instructions could be made easier or more friendly; and 2 students (3%) thought that cash would be beneficial as an additional payment option for in-app goods and services.

## Discussion

In this study of the utilization of a mobile phone app to increase access to SRH information, goods, and services among university students in Uganda, utilization (81%) was high. The utilization was unrelated to any participant characteristics, including gender. The utilization of the app was frequent, with 70% of participants using the app at least once a month over the six month period, but was hampered by technical issues such as internet speeds and app glitches. The most popular information portal was the FAQs, and the most popular service was HIV testing and counseling. The most popular goods were condoms for males and sanitary pads for females.

The utilization of the MPA over the six-month period was high (81%). The utilization was frequent, suggesting ongoing engagement by app users and uniform across the information, goods, and services dimensions. Further still, there was lower utilization of the app once downloaded (81%) compared to acceptance of the app for download (86%), suggesting a difference in app acceptance and downstream acceptance of services [23]. The utilization was hampered by technical issues and glitches. Although technical challenges have been reported in other mHealth studies [13], they were expected, given that the app was a prototype developed within a short period of time. In response to the results of the pilot test, including insights from the utilization of the app, our team of investigators intends to improve the prototype with a view to fixing technical glitches and increasing the rate of app utilization and engagement once downloaded.

mHealth interventions, particularly using text messaging, used predominantly for the provision of information, but also increasingly for connecting clients to services, are increasing in low-income settings [13]. In addition to providing access to information, our app based SRH intervention provided an interface to access goods and services by enabling links to providers, transporters, and a payments portal. This allowed access to information, goods, and services while assuring privacy and confidentiality, and enabling convenience by easing transportation and payment. For the section of the population with internet-enabled mobile phones that can download and use apps, our intervention has the potential to improve SRH care delivery in Uganda and other low-income settings [27].

The most popular information portal (FAQs), goods (sanitary pads and condoms), and services (HIV testing and counseling) are all in constant demand. This suggests that solving technical problems will have a high likelihood of creating an app that can be used on an ongoing basis. Other information portals (e.g., chatbox), goods (e.g., pregnancy tests), and services (e.g., STI diagnosis and treatment) may be required once in a while, but their availability reduces delays in access and increases prompt clinical management, thereby improving health outcomes. The app, therefore, fulfills both routine and urgent SRH needs in this population and approaches comprehensive SRH care, excluding care for emergencies.

In addition to high utilization, the MPA and related services demonstrated good functionality attributes, including good provider responsiveness through the GHE clinic representative management dashboard within the MPA and ease of payments. Notably, a majority of participants did not find the in-app advertisements distracting. This may have been responsible for the perception by participants of the usefulness of the MPA and the need for it to be disseminated among other students. It also bodes well for the potential success of the transition to scale and commercialization of this intervention; in-app advertising will generate revenue for subsidies, planned for low-income users, and operations. There was, however, consistency in the view by participants of the presence of significant technical glitches in the MPA and the perception that some technical aspects of the MPA needed improvement. Before the transition to scale, widespread dissemination of the MPA, and commercialization, technical improvements will be needed as well as improvement in MPA-related services such as responsiveness of providers to demand for goods and services, increased payment options, and the overall MPA feeling and user experience.

Our study was cross-sectional in nature and says nothing about the ongoing utilization of the MPA beyond a six-month period. Additionally, the MPA was exclusive to the Android platform, which despite being the most common in Uganda, excludes the users of other platforms. Future iterations of the MPA will be developed using other mobile platforms to enable uniform access. Although we expect improved versions of the app in the future to be marginal and to improve on the current version, these data are only relevant to the version that was pilot tested. Acceptability and utilization will be subject to an ongoing assessment, even when we implement the intervention at scale as planned.

## Conclusion

Along the continuum of innovation in healthcare, the utilization of services is key. This study demonstrates that university students in Uganda find a mobile phone application to increase access to SRH information, goods, and services acceptable and usable. Given the effectiveness of the MPA in increasing access to SRH information, goods, and services (27), this study provides the basis for further improvement of both the evidence base (economic efficiency and scalability) and technical features of the app for use in this population, and potentially, for scale up to other populations with access to internet-enabled mobile phones, to improve SRH in Uganda and other low-income settings.

## Data Availability

All data used in this study will be provided upon request.

## Abbreviations

CIs: Confidence intervals
DHIs: Digital health interventions
FAQs: Frequently asked questions
GCC: Grand Challenges Canada
KYU: Kyambogo University
mHealth: Mobile health
MPA: Mobile phone application
ODK: Open data kit
RCT: Randomized controlled trial
SRH: Sexual and reproductive health
STIs: Sexually transmitted infections
